# Lesion-Based Bone Metastasis Detection in Chest Bone Scintigraphy Images of Prostate Cancer Patients Using Pre-Train, Negative Mining, and Deep Learning

**DOI:** 10.3390/diagnostics11030518

**Published:** 2021-03-15

**Authors:** Da-Chuan Cheng, Te-Chun Hsieh, Kuo-Yang Yen, Chia-Hung Kao

**Affiliations:** 1 Department of Biomedical Imaging and Radiological Science, China Medical University, Taichung 404, Taiwan; 2 Center of Augmented Intelligence in Healthcare, China Medical University Hospital, Taichung 404, Taiwan; D10119@mail.cmuh.org.tw; 3 Department of Nuclear Medicine and PET Center, China Medical University Hospital, Taichung 404, Taiwan; T10540@mail.cmuh.org.tw

**Keywords:** bone metastasis detection, classification, YOLO, pre-train, negative mining, transfer learning, deep learning

## Abstract

This study aimed to explore efficient ways to diagnose bone metastasis early using bone scintigraphy images through negative mining, pre-training, the convolutional neural network, and deep learning. We studied 205 prostate cancer patients and 371 breast cancer patients and used bone scintigraphy data from breast cancer patients to pre-train a YOLO v4 with a false-positive reduction strategy. With the pre-trained model, transferred learning was applied to prostate cancer patients to build a model to detect and identify metastasis locations using bone scintigraphy. Ten-fold cross validation was conducted. The mean sensitivity and precision rates for bone metastasis location detection and classification (lesion-based) in the chests of prostate patients were 0.72 ± 0.04 and 0.90 ± 0.04, respectively. The mean sensitivity and specificity rates for bone metastasis classification (patient-based) in the chests of prostate patients were 0.94 ± 0.09 and 0.92 ± 0.09, respectively. The developed system has the potential to provide pre-diagnostic reports to aid in physicians’ final decisions.

## 1. Introduction

According to a report published in 2018 by the National Health Insurance Research Database of Taiwan, prostate cancer (PC) is the seventh highest ranking cause of cancer-related deaths among Taiwanese men [1]. PC involves a high degree of osteotropism [2] because the possibility of metastases is relatively high. However, PC has a slower rate of progression than many other cancers. According to one report by the American Cancer Society, if PC has only spread to the bones and not to other organs, radium-223 can be used to help people live longer [3]. If the cancer has grown outside the prostate, preventing or slowing the spread of the cancer to the bones is a major treatment goal. If the cancer has already reached the bones, controlling or relieving pain and other complications is an important part of treatment. As mentioned in [4], “the choice of treatment strategy is influenced by the presence or absence of bone metastases”, so early diagnosis is clinically important. The 5-year relative survival rate for individuals with PC that has spread to distant lymph nodes, organs, or the bones is 29% [3]. Patients with only bone metastases can be treated with hormone therapy, chemotherapy, or radiation therapy. Early identification of PC metastases is important, because therapy can effectively slow metastasis progression at this stage. One of the primary imaging techniques used in clinics for bone metastasis diagnosis is the whole-body bone scan (WBBS) with vein injection using the Tc-99m MDP tracer [5,6]. WBBS is also known as bone scintigraphy (BS). Many imaging modalities were surveyed by [4], including BS, X-radiography (XR), computed tomography (CT), magnetic resonance imaging (MRI), positron emission tomography (PET), and single photon emission computed tomography (SPECT). Among these modalities, BS provides a trade-off between diagnostic efficiency and cost, and it is “the most commonly used means of detecting bone metastasis” [4]. In 2021, the cost of BS examination in Taiwan was about USD 108 [7], which is lower than the same examination at a hospital in US. The estimated national average price of BS in the US is about USD 1217, according to the MDsave’s website [8].

Based on BS, many researchers have developed CAD (computer-aided diagnosis) methods to aid in bone metastasis detection or classification. In general, these can be categorized into three types: (1) classification of the BS if it is bone metastasis (patient-based); (2) detection and classification of hot spots (lesion-based); and (3) calculation of the BSI (bone scan index). From a technical difficulty viewpoint, this gradually increases as the type number increases. For the first type, it is only necessary to know whether one patient has the bone metastasis or not [9,10,11]. For the second type, hot spots must be detected and classified correctly. For the third type, it is necessary to detect the hot spots, classify them, and detect the bone area related to the calculation of the bone scan index (BSI) [12,13].

One biomarker used to measure the level bone metastasis invasion is the bone scan index (BSI). The BSI was proposed in 1998 [14]. A US patent related to the BSI was issued in 2012 [15]. The publication related to this patent is [16]; however, no description of the measurement technique is provided in the publication. We only know that in [16], the authors extracted 20–30 hotspot features and used a fully connected NN (Neural Network) as a classifier. They used 795 patients as the training group, and >40,000 hotspots were collected for various metastatic cancers (e.g., prostate, breast, and kidney cancers). The system used in [16] detected hotspots suitably in certain areas; however, it could not detect hotspots in a large area of bone metastasis (Figure 3 in [16]). The reason for this result might be that there were limited training data available on hotspots. In [12], the authors used ResNet50 as a backbone and incorporated the ladder network to form an LFPN (ladder feature pyramid network), which can use unlabeled data to pre-train the NN for bone metastasis detection in the chest area. The mean sensitivity and precision of lesion detection were 0.856 and 0.852, respectively. For metastasis classification in the chest, the sensitivity and specificity were 0.657 and 0.857, respectively. The aforementioned study provided useful technical details on metastasis detection and classification using deep learning.

Some strategies are able to increase performance by using CNN and deep learning, as follows:

(1) By using a pre-trained model, also known as transfer learning [17].
(2) By using data augmentation.
(3) By using an ablation study to find a near-optimal hyper-parameter set.
(4) By using hard negative mining to reduce the false positive rate.
(5) By using different CNN backbones and finding the best one.
(6) By using image enhancement as a pre-process before inputting images into CNN.

Deep learning can perform well when the training dataset is large. However, in some medical studies, small datasets have been used, like in this study. Under this situation, traditional image enhancement might play a crucial role in increasing the NN’s performance and robustness.

In general, there are four types of object detection and semantic segmentation methods used in computer vision. From coarse to fine, these are (1) classification, (2) classification and localization, (3) object detection, and (4) instance segmentation. The classification only needs to determine the class of an image. Many CNNs can fulfill the requirement. Localization needs to be used to locate the exact position of the object in an image using a bounding box in the tightest possible manner. Object detection can detect multiple objects of different classes in an image using some bounding boxes to denote their locations, such as the YOLO [18,19] series and faster R-CNN [20]. Instance segmentation methods, such as mask R-CNN, do the same thing but use the finest option, a mask, to identify the boundary of each object instead of using a bounding box [21]. Moreover, the difference between instance segmentation and semantic segmentation is that the former is able to differentiate between different objects of the same class. In computer vision, this is done by machine learning with some hand-crafted features, as shown in previous studies [22,23]. In this paper, we use the object detection method YOLO v4 to identify the locations of lesions and classify them into two classes: metastasis or not.

The YOLO (You Only Look Once) method is a state-of-the-art, real-time object detection system [24]. As the authors claim, “It (YOLO v3) also makes predictions with a single network evaluation unlike systems like R-CNN which require thousands for a single image. This makes it extremely fast, more than 1000× faster than R-CNN and 100× faster than Fast R-CNN”. Basically, YOLO v3 contains a backbone (here, Darknet-53) for feature extraction and a region proposal network (RPN). The input is an image, and the outputs are bounding boxes (with centroid coordinates, widths, and heights). Each box has a classification label and a probability (or named as confidence). YOLO v4, its updated version [19], runs faster and has a better performance than earlier versions.

## 2. Materials and Methods

### 2.1. Materials

In this retrospective study, 576 WBBS images were collected from China Medical University Hospital between August 2013 and May 2019, of which 205 came from PC patients and 371 came from breast cancer patients. This study was approved by the Institutional Review Board (IRB) of China Medical University and Hospital Research Ethics Committee (CMUH106-REC2-130). The first IRB has been approved in 27 September 2017. The collected WBBS images were in DICOM format, and all private connections were removed. The spatial resolution of the raw images was 1024 × 512 pixels (a combination of the anterior–posterior (AP) view and the posterior–anterior (PA) view). The intensity information from each pixel was saved as files of 2 bytes in size (int16).

The WBBS process can be described as follows. Patients underwent whole-body planar bone scans (WBBS) with a gamma camera (Millennium MG, Infinia Hawkeye 4, or Discovery NM/CT 670 system; GE Healthcare, Waukesha, WI, USA). Bone scans were acquired 2–6 h after the intravenous administration of 20 mCi of technetium-99m methylene diphosphonate (Tc-99m MDP) by using a low-energy, high-resolution or general-purpose collimator with a matrix size of 1024 × 256, an acquisition time of 15–20 cm/min, and photon energy centered on the 140-keV photo-peak with a symmetrical 20% energy window. During the wait time, immediately before scanning, the patients were encouraged to hydrate and void frequently. The patients were scanned in the supine position within 15 min and whole-body anterior–posterior images were acquired for interpretation. All images were interpreted using a dedicated GE Xeleris workstation (GE Medical Systems, Haifa, Israel; version 2.0551).

All images were studied by two experienced physicians. Hotspots were categorized into two types: (1) confirmed metastatic (or positive) hotspots or (2) equivocal or normal lesions (including degenerative changes and inflammation) and injuries (post-trauma). Positive hotspot classification was confirmed and agreed upon by two experienced nuclear medicine physicians according to pathological examination results, relevant medical history, characteristic findings on other advanced medical imaging modalities (e.g., computed tomography or magnetic resonance image), and/or serial changes on follow-up bone scans. Equivocal hotspots were those lacking definite evidence or for which agreement from the two experienced physicians did not occur. Of the 205 PC patients, 11 were excluded because of superscans. The remaining number of PC patients used in this study was 194. The PC patients were aged between 51 and 92 years, and the average age was 73.9 ± 8.3 years. The 371 breast cancer patients were only used in the pre-training period. The number of metastases identified in PC images was 524, the number of equivocal and injury hotspots was 103, and the number of normal hotspots was 198 (Table 1). For the two-level classification, class 0 denoted metastasis and class 1 denoted equivocal, injury, and normal hotspots.

### 2.2. Network Selection: YOLO v4

The difficulty with automatic bone metastasis detection comes from the differentiation between normal and metastatic hotspots because injury and osteoarthritis may also cause hotspots. However, well-trained physicians have the ability to differentiate between them. For example, injury hotspots may occur in not only one spot but along a straight line (on the ribs). Osteoarthritic hotspots might be symmetric on both sides (left and right) near a joint. Human experts use certain knowledge to recognize and differentiate between hotspots. Such knowledge is non-trivial for mathematical modeling and is embedded into algorithms used in traditional image processing techniques.

The CNN has been used for more than 10 years to extract features [25]. The NN provides an alternative method to extract non-handcrafted features automatically via a training process. There are two state-of-the-art networks that are able to detect and classify multiple objects in an image using bounding boxes: (1) Faster R-CNN [20]; (2) YOLO v3 [18,24], and YOLO v4 [19]. The major differences between these two models are that the Faster R-CNN is a two-stage model, while YOLO v3 (and v4) is a one-stage model. As stated in [24], the speed of YOLO v3 is 100 times faster than that of the faster R-CNN. Further, YOLO v4 [19] performs better than YOLO v3 [18]. Thus, we chose to use YOLO v4 in this study. “It is very hard to have a fair comparison among different object detectors”. As the author Jonathan Hui said in his personal medium in 2018 [26], “There is no straight answer on which model is the best”. What we selected is only a trade-off between accuracy and speed, since there are too many parameters impacting the performance. As shown by the figures in [26] selected from some papers, YOLO v4 is not a bad choice. In fact, YOLO v4 is the most updated and state-of-the-art technique found in similar works.

### 2.3. Image Pre-Processing

Image size and intensity normalization is an important step that must be done prior to image processing. The acquired WBBS images had large variations in intensity distribution. These variations were caused by many factors, such as the blood supply to the bones, the drug metabolism rate, and leakage of the radiotracer. Most our WBBS images were of a consistent quality, but some were not. To alleviate this problem, we propose the use of an automated image normalization strategy, as detailed in the following two paragraphs:

A standard WBBS image has two views: anterior and posterior. The body range was detected using projection profiles, and both views were cut and centered into an image with a size of 512 × 950 pixels without scaling or any other geometric transformation. Until this point, the image was still in int16 format.

**Figure 1:**
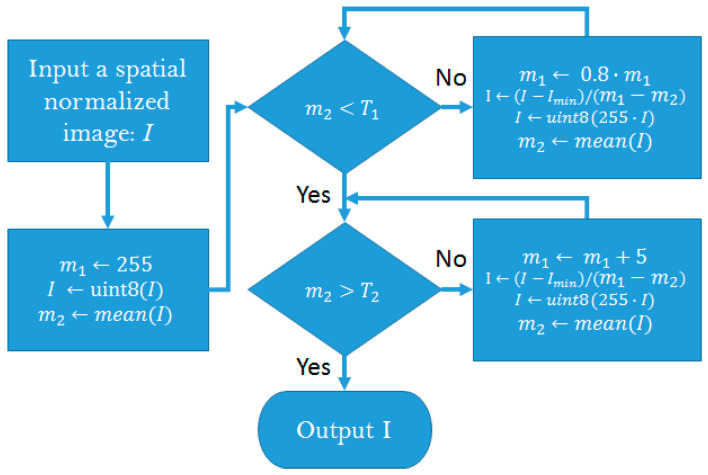
Flowchart of intensity normalization. During this process, intensities greater than 255 were pruned via the uint8 function. Notably, image *I* was converted to double precision before computation in every process block.

The intensity of the WBBS images revealed the absorption of Tc-99m MDP on the gamma camera. Some special cases, such as leakage of the radiotracer and from the urine bag (usually near the femur), also caused high intensity in the image. Our strategy to solve this problem was to design an algorithm that controlled the average intensity within a range (T_1_, T_2_), as follows (Figure 1):

We developed a network to detect the chest and pelvis areas of the bone scan images in our previous study [27]. This network extracted the chest images for further processing in this study.

### 2.4. Data Augmentation

When we train a neural network, what we are really doing is tuning the parameters (weights between neurons) such that the network can fit a particular input (an image) to its output (a label). Typical state-of-the-art networks have parameters in the order of millions. If there are not enough training images, most parameters are under-trained, so the performance of the network might be poor. One intuitive solution is to increase the number of training images; this is why data augmentation is done. A good review of data augmentation can be found in [28].

We performed data augmentation offline on extracted chest images, as follows: (1) we generated 6 different intensity images; (2) we flipped every image. Thus, the number of items in the dataset was increased by a factor of 12. More specifically, we found that the average intensity of the chest images needed to be controlled between (25, 48). For images under the lower mean of 25, some parts of ribs might disappear. For images over the upper mean of 48, some images might be similar to superscans, causing ambiguity in hotspot detection. The average intensity of the first image was counted, and then the extra 6-intensity images were examined. Their average intensities were found to be uniformly distributed within the range (25, 48).

### 2.5. Input Image

**Figure 2:**
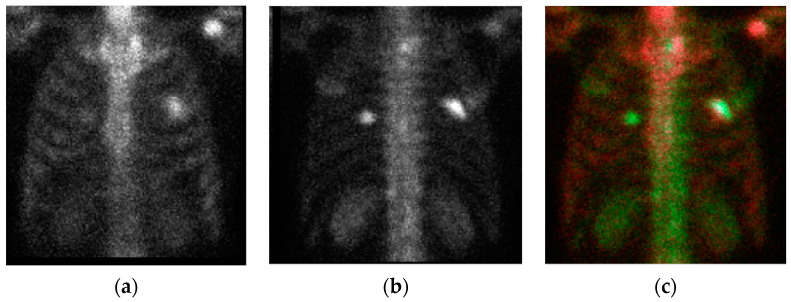
The chest images. (**a**) anterior–posterior (AP) view; (**b**) flipped posterior–anterior (PA) view; (**c**) the combined chest color image for input into the NN (neural network).

The raw images had AP and PA views. If we separated them or fed them in a plane as previous studies did, then we lost their corresponding position connections. To alleviate this problem, we combined them to form a 3D image to be the input of the YOLO v4 network. The PA view was flipped (left-right) to be the ‘green’ channel, whereas the AP view was inserted to be the ‘red’ channel. A third image was produced by multiplying the AP and PA views pixel-by-pixel, and then the average intensity of the third image was controlled to be the mean of these two views. In this way, we produced a color image, as shown in Figure 2.

### 2.6. Pre-Trained Model and Transfer Learning

Transfer learning is a strategy that is commonly used to increase the performance of a NN; however, it is only helpful if the learned images are similar to the test data. The COCO dataset contains nature pictures that are different to bone scan images; therefore, transfer learning from the pre-trained model is useless. 

In this study we collected 371 WBBS images from breast cancer patients and used them to train a model. All 371 WBBS images were pre-processed, as described in Section 2.3, Section 2.4 and Section 2.5. Among these 371 images, 167 images showed bone metastases and 204 images were normal without metastasis or injury. The metastatic hotspots in these 167 images were manually extracted by an expert using bounding boxes. We trained a YOLO v4 model with only one class, metastasis, using these 167 images with their corresponding labels (bounding box: x, y, width, height). We describe how this pre-model was further trained using negative mining in the next paragraph.

### 2.7. Negative Mining

The current YOLO v4 pre-model was able to detect hotspots, both normal and abnormal. We then applied this pre-model to test those 204 normal images (without metastasis) to produce false positives, intentionally. This is similar to the hard negative mining process; however, it is not hard. Since we knew that all 204 images were normal, all resulting bounding boxes were false positives (namely, negatives). Then, all of these false positives and previous true positives were fed into the trained YOLO v4 model and trained again to get a pre-trained model with two classes. By using this strategy, we did not need to prepare any negatives for training purposes manually. Thus, the process was efficient and saved a tremendous amount of time. At this point, there were two classes in the pre-trained model: (1) metastasis; (2) non-metastasis.

Hard negative mining is a way to explore hard negatives using a current model and then training the model again with the explored hard-negatives and old training samples. The model with hard negatives would be expected to perform better [26]. In this study, we used this idea to produce many training samples of a new class fully automatically. The goal was to reduce the false positive rate of the pre-trained model, as hard negative mining does.

### 2.8. Transfer Learning

The pre-trained model was further trained by the data of PC patients’ bone scan images. All 194 PC bone scan images were processed through the methods described in paragraphs 2.3 to 2.5. Three PC data classes were used: (1) Metastasis; (2) Equivocal; (3) Injury and other normal hotspots. There were less images in class 2 than in classes 1 and 3.

We used 10-fold cross validation. The metastasis images were randomly shuffled 10 times, and each time, one-tenth of these were used for testing and nine-tenths were used for training. The same thing was done with the normal (with injury) images. The number of equivocal cases was very limited, and these images were put in the normal group.

## 3. Results

**Figure 3:**
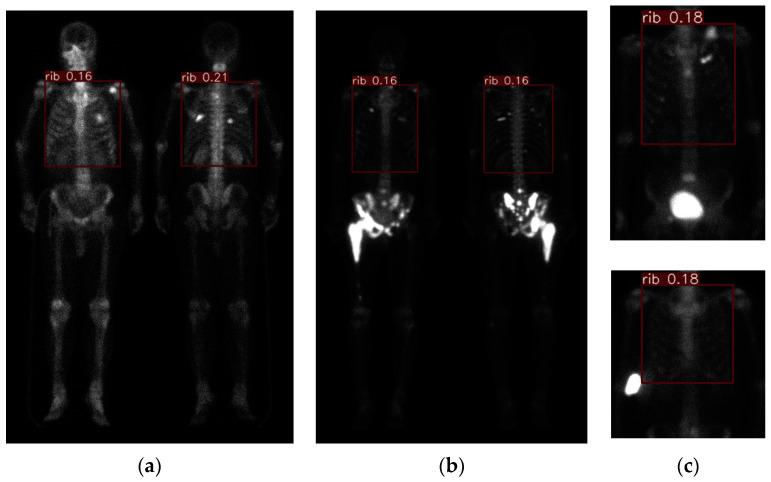
Without a loss of generality, several extreme results of image pre-processing are shown. (**a**) result (case number 9) representing most cases; (**b**) pelvic metastasis with strong Tc-99m MDP absorption (case number 13); (**c**) urine remaining in the bladder (case number 71) and injection leakage (case number 60).

Figure 3a demonstrates the result of the pre-processing (Section 2.3, (T_1_, T_2_) = (7,14)) and chest region detection (method described in our previous study [24]). The chest regions were combined to form a three-channel color image, as described in Section 2.5, and the results are shown in Figure 2. To provide a comprehensive overview, we show an extreme case in Figure 3b. In Figure 3b, we can see that the chest region has a very low density compared with Figure 3a. This is because image pre-processing was done before chest detection and was applied to the whole body including the pelvis. Once the pelvis has metastases, the average intensity will be affected by this part, and the intensity in the rest of the image will be suppressed. This is also why we controlled the average intensity of the chest region in the range of (25, 48). A similar effect occurs in cases such as injection leakage and urine, as shown in Figure 3c.

**Figure 4:**
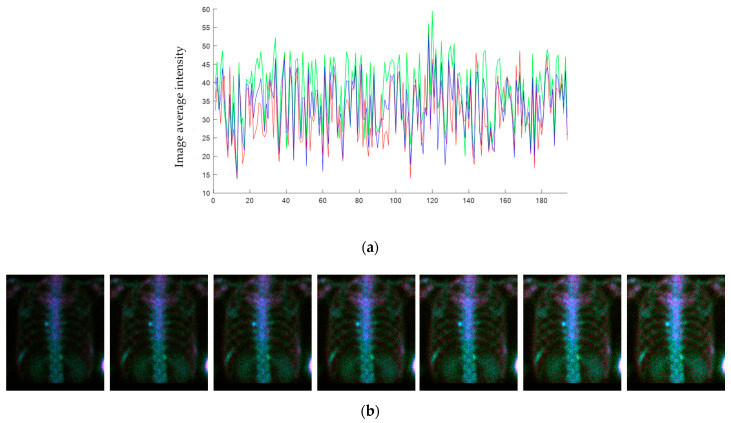
(**a**) The average intensity of each chest image. The color denotes the corresponding channel in the color image, as shown in Figure 2c. The abscissa shows the case numbers; the ordinate shows the average image intensities. (**b**) The 7 different intensities were augmented as an illustration. (Case number 07).

Figure 4 shows the average intensity of the chest images before data augmentation. The abscissa is the case number of PC patients, whereas the ordinate is the average intensity of an image. Most cases were within the intensity range (25, 48). After data augmentation, each chest image had six different intensity levels that were uniformly distributed within the given range.

A YOLO v4 model was trained with 167 bone metastasis images of breast cancer patients. In this stage, there was only one class, metastasis, and we stopped after 150,000 iterations to build a pre-model. The batch size was 64, and the learning rate was 0.00261. The pre-model was used to detect hotspots in the 204 normal cases. Since the model was trained with only one class, it could only detect one class. We set the confidence threshold at 0.1, and all results greater than that threshold were collected to form negatives. These negatives were used as training samples of the second class: non-metastasis. The pre-model was further trained with these negatives and together with former positives to build a pre-trained model.

**Figure 5:**
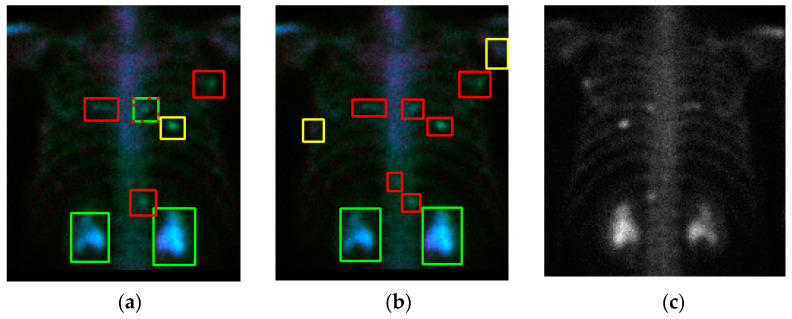
The qualitative result (case number 63) of our model. (**a**) Detection and classification results; (**b**) ground truth; (**c**) the raw PA view (without flipping). The red box denotes metastasis, the yellow box denotes equivocal, the green box denotes the normal or injury. The green box with red dots denotes this ROI has two class probabilities: however, solid box has a larger probability than doted box has. The same meanings are in the following figures.

Cross-validation is a technique that is used to evaluate a model by partitioning the original sample into a training set to train the model and a test set to evaluate it. The PC images were randomly partitioned into 10 subsamples of equal size by using the ‘shuffle’ command. Of the 10 subsamples (or named as 10 folds), one single subsample (1 fold) was retained as the validation data for testing the model. The remaining 9 subsamples (9 fold) were used as training data. The cross-validation process was then repeated 10 times. Figure 5a shows a qualitative detection and classification result. In the following similar figures, the three classes (metastasis, equivocal, non-metastasis) are denoted by three colors (red, yellow, green). In Figure 5a, there is a green box with red dots. This means that there are two classes (meta and non-meta) detected with overlapping. In the overlapping cases, if the confidence level is larger, it is represented as a line; otherwise, it is shown with dots. Figure 5b shows the ground truth, and Figure 5c shows the corresponding PA view without flipping. In this case, some “under-diagnosis” can be observed.

**Figure 6:**
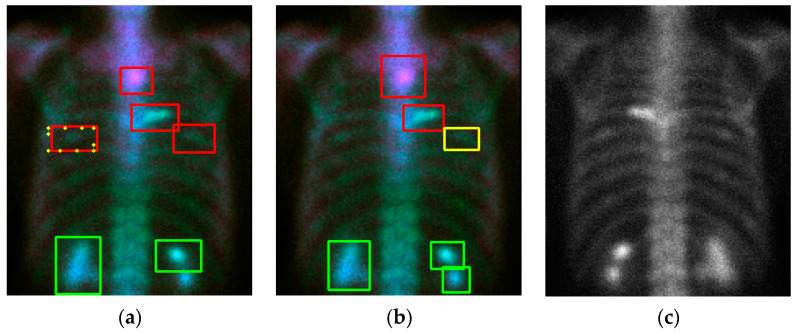
The qualitative results (case number 125) of our model. (**a**) Detection and classification results; (**b**) ground truth; (**c**) the raw PA view (without flipping).

**Figure 7:**
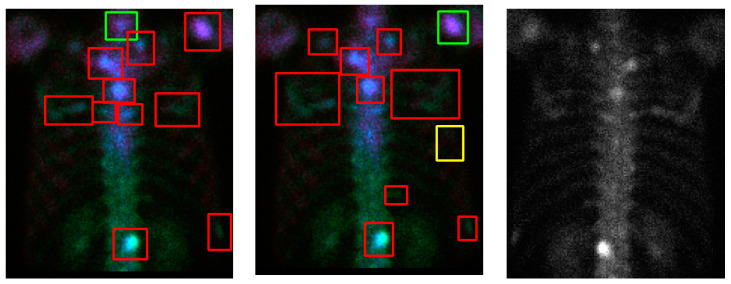

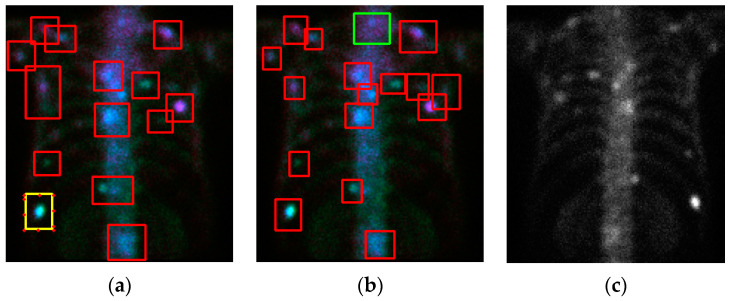
Qualitative results (case number 85, upper row; case number 105, lower row) of our model. (**a**) Detection and classification results; (**b**) ground truth; (**c**) the raw PA view (without flipping).

Without losing generality we show some examples in Figure 6 and Figure 7. In the case of Figure 6, e the model shows some instances of “over-diagnosis” different to those shown in Figure 5. The physicians were not sure about a region (shown in Figure 6b, yellow box) and gave an equivocal decision, whereas the model classified it as a “meta”. Another region was marked by the model with a red line and yellow dots, but this region was ignored by physicians. Figure 7 demonstrates cases with multiple metastases. There were three false negatives in case number 105.

**Figure 8:**
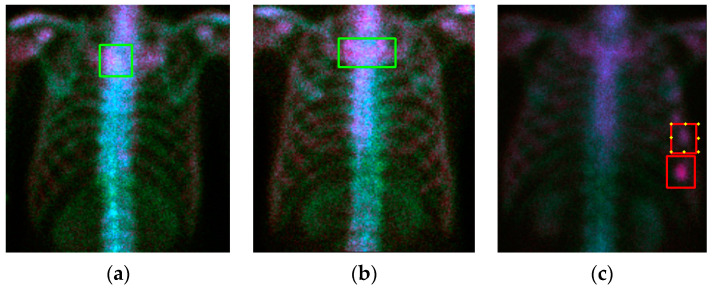
Qualitative detection and classification results (case numbers 73, 132, and 137). (**a**) Normal case #73; (**b**) normal case #132; (**c**) case #137 with injuries.

The readers might wonder how this model works in normal cases with a high image intensity. Figure 8a,b shows two examples of this case. Our model works very well without error. However, Figure 8c shows an injury case. In this case, the model miss-classified two injury hotspots as metastases, although one of them had the possibility of being equivocal.

We further show the quantitative results of YOLO v4 in Table 2. The metastasis and normal cases were controlled so that they averaged in 10-fold. Among them, the images of nine folds were used to train the pre-trained YOLO v4 and the images of one fold were used for testing. In the lesion-based experiment, each detected bounding box was compared with the ground-truth determined by two physicians using the 0.3 IoU (intersection of union). We considered two classes: metastasis and non-metastasis. The equivocal cases were ignored. We were able to calculate the sensitivity and precision for the lesion-based case. This was because the term true-negative (TN) has no definition. Therefore, only ‘precision’ can be calculated, which is defined by (TP)/(TP + FP). In the patient-based experiment, we only considered whether the chest images showed metastasis or not. Thus, the term true negative could be defined. Therefore, we could calculate the sensitivity and specificity.

To compare our model with another similar state-of-the-art network, we used the faster R-CNN [20], and the results are shown in Table 3. The comparison was based on the same training and test samples, and these samples were pre-trained by the same breast dataset. Table 2 and Table 3 show that the YOLO v4 is more advanced than the Faster R-CNN.

**Figure 9:**
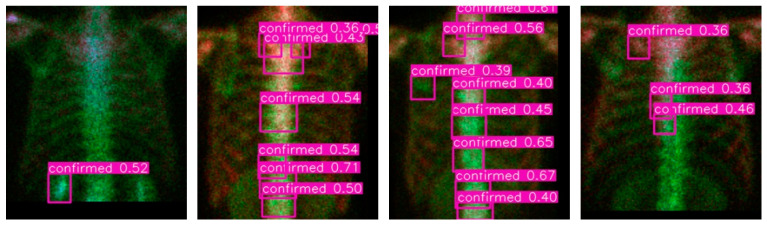
Qualitative results of the negative mining process on the breast cancer patients without metastases on the chest.

We report some details of the negative mining. In total 371 breast patients bone scan images are involved in the pre-train and negative mining processes. In which 167 cases have chest metastases and the rest 204 cases are normal in chest. By using the 1-class (metastasis) training, we obtain an NN model that can only recognize positives. Use this model to detect in the 204 normal cases we have mined 746 negatives in total, which are turned to be the third class (normal) to get a pre-train model together with previous positive samples. Figure 9 show four qualitative results of the negative mining. In the figures, ‘confirmed’ means the ‘confirmed metastasis’, and the number aside it is the detected confidence (the probability). Via this strategy, we only need to label positives and let the negatives be mined. This process will save time and get efficient training samples.

## 4. Discussion

In the field of computer vision, comparison between different methods using the same benchmark is important. However, there are no open datasets for bone scan images, unlike the lung nodule detection, for which CT images are available. The well-known LUNA2016 dataset [27] is a selected subset of the LIDC-IDRI [28], which contains CT image sequences from 888 patients. These open datasets are benchmarks for the comparison of different methods. In bone scan metastasis detection and classification research, all previous studies have used in-house datasets of a gold standard. This makes the comparison of different algorithms difficult, especially for works that did not provide the source codes. We were not able to try other datasets using our algorithm. Therefore, the performances reported by other researchers can only be used as references, rather than for objective comparison.

Similar research was reported in [12] in 2020. The authors used a ladder network to pre-train an NN backbone with an unlabeled dataset. The pre-trained model worked better than the one without pre-training. For lesion detection, the mean sensitivity and precision values were 0.856 and 0.852. However, this was the only detection method without classification. Our results showed sensitivity and precision values of 0.72 and 0.90, and this indicated not only correct detection but also correct classification. In the previous study, for metastasis classification in the chest, the sensitivity and specificity values were 0.657 and 0.857, respectively. In our study, the sensitivity and specificity values for chest image metastasis classification were 0.94 and 0.92, respectively.

To the best of our knowledge, this is the first study to propose the use of negative mining to prepare training patterns of another class in order to reduce the false positive rate. Our idea is that since we do not know what false positives the model will produce, we should let the model tell us. We just select some negative cases for the model to test and collect all of the results as false positives in the next training phase. Thus, we save a tremendous amount of time in preparing training patterns. Via this strategy, the rate of false positives obviously reduces. We provide this idea for other researchers and hope it is helpful for future study.

Some parameters used in data augmentation, such as zoom in, zoom out, and rotation can increase the robustness of a neural network. We did not implement them in this study due to the computational cost involved—the training (150,000 iterations) of one fold takes more than 8 h in our DGX-2 station.

In this study, we did not conduct an ablation study to find a near-optimal hyper-parameter set. For example, we did not determine the optimal learning rate at the beginning or the optimal decay rate of the learning rate. We think that this depends on training data and it is not necessary to explore it because of the associated computation cost. We just leveraged the hyper-parameters, as suggested by the original authors.

## 5. Conclusions

We provide an efficient way to reduce the false positive rate by using negative mining. According to our experiments using 10 shuffles with 10-fold cross validation, the detection and classification of metastasis hotspots has mean sensitivity and precision values of 0.72 and 0.90, respectively. Chest image classification has mean sensitivity and specificity values of 0.94 and 0.92, respectively. The higher precision and specificity values indicate a reduced false positive rate.

## Figures and Tables

**Table 1:** The number of (a) metastatic lesions, (b) equivocal, injury, and (c) degenerative changes, and normal hotspots such as urine in the bladder, hotspots in the kidneys, etc. in 194 PC BS images.

Metastasis	Equivocal and Injury	Normal Hotspots
524	103	198

**Table 2:** The quantitative results of the YOLO v4. All 194 prostate cancer (PC) bone scintigraphy (BS) images were shuffled 10 times. One shuffle means one cross-validation.

Shuffle Number	Lesion-Based Sensitivity Precision		Patient-Based Sensitivity Specificity	
1	0.69	0.83	1.00	0.83
2	0.70	0.86	0.75	0.91
3	0.68	0.87	0.88	0.73
4	0.68	0.97	0.88	1.00
5	0.71	0.94	1.00	1.00
6	0.70	0.92	1.00	1.00
7	0.77	0.87	1.00	0.79
8	0.71	0.92	1.00	1.00
9	0.73	0.91	1.00	0.91
10	0.78	0.94	0.88	1.00
Average	0.72 ± 0.04	0.90 ± 0.04	0.94 ± 0.09	0.92 ± 0.09

**Table 3:** The quantitative results of the Faster R-CNN. All 194 PC BS images were shuffled 10 times. One shuffle means one cross-validation.

Shuffle Number	Lesion-Based Sensitivity Precision		Patient-Based Sensitivity Specificity	
1	0.77	0.66	1.00	0.90
2	0.67	0.59	1.00	0.70
3	0.75	0.68	1.00	0.60
4	0.65	0.50	0.86	0.36
5	0.68	0.59	0.88	0.64
6	0.74	0.67	0.88	1.00
7	0.56	0.78	1.00	0.73
8	0.70	0.54	0.88	0.82
9	0.72	0.74	0.89	0.70
10	0.48	0.77	0.75	0.82
Average	0.67 ± 0.09	0.65 ± 0.10	0.91 ± 0.08	0.73 ± 0.18

## Data Availability

Not applicable.

## References

[1] National Health Insurance Research Database. https://www.mohw.gov.tw/cp-4256-48057-1.html.

[2] Bubendorf L., Schöpfer A., Wagner U., Sauter G., Moch H., Willi N., Gasser T.C., Mihatsch M.J. (2000). Metastatic patterns of prostate cancer: An autopsy study of 1589 patients. Hum. Pathol..

[3] The American Cancer Society Medical and Editorial Content Team Treating Prostate Cancer Spread to Bones. https://www.cancer.org/cancer/prostate-cancer/treating/treating-pain.html.

[4] Sadik M., Hamadeh I., Nordblom P., Suurkula M., Höglund P., Ohlsson M., Edenbrandt L. (2008). Computer-assisted interpretation of planar whole-body bone scans. J. Nucl. Med..

[5] Hamaoka T., Madewell J.E., Podoloff D.A., Hortobagyi G.N., Ueno N.T. (2004). Bone imaging in metastatic breast cancer. J. Clin. Oncol..

[6] Even-Sapir E., Metser U., Mishani E., Lievshitz G., Lerman H., Leibovitch I. (2006). The detection of bone metastases in patients with high-risk prostate cancer: 99mTc-MDP Planar bone scintigraphy, single- and multifield-of-viewSPECT, 18F-fluoride PET, and 18F-fluoride PET/CT. J. Nucl. Med..

[7] Medical Service Payment Items and Payment Standards, National Health Insurance of Taiwan, ROC. https://www.nhi.gov.tw/Content_List.aspx?n=58ED9C8D8417D00B.

[8] How Much Does a Bone Scan Cost? MDsave. https://www.mdsave.com/procedures/bone-scan/d784f4c8.

[9] Panpandrianos N., Papageorgiou E., Anagnostis A., Papageorgiou K. (2020). Bone metastasis classification using whole body images from prostate cancer patients based on convolutional neural networks application. PLoS ONE.

[10] Wuestemann J., Hupfeld S., Kupitz D., Genseke P., Schenke S., Pech M., Kreissl M.C., Grosser O.S. (2020). Analysis of bone scans in various tumor entities using a deep-learning-based artificial neural netowk algorithm-Evaluation of diagnostic performance. Cancers.

[11] Zhao Z., Pi Y., Jiang L., Xiang Y., Wei J., Yang P., Zhang W., Zhong X., Zhou K., Li Y. (2020). Deep neural network based artificial intelligence assisted diagnosis of bone scintigraphy for cancer bone metastasis. Sci. Rep..

[12] Apiparakoon T., Rakratchatakul N., Chantadisai M., Vutrapongwatana U., Kingpetch K., Sirisalipoch S., Rakvongthai Y., Chaiwatanarat T., Chuangsuwanich E. (2020). MaligNet: Semisupervised learning for bone lesion instance segmentation using bone scintigraphy. IEEE Access.

[13] Shimizu A., Wakabayashi H., Kanamori T., Saito A., Nishikawa K., Daisaki H., Higashiyama S., Kawabe J. (2020). Automated measurement of bone scan index from a whole-body bone scintigram. Int. J. Comput. Assist. Radiol. Surg..

[14] Imbriaco M., Larson S.M., Yeung H.W., Mawlawi O.R., Erdi Y., Venkatraman E.S., Scher H.I. (1998). A new parameter for measuring metastatic bone involvement by prostate cancer: The Bone Scan Index. Clin. Cancer Res..

[15] (2015). Computer-Aided Bone Scan Assessment with Automated Lesion Detection and Quantitative Assessment of Bone Disease Burden Changes. U.S. Patent.

[16] Ulmert D., Kaboteh R., Fox J.J., Savage C., Evans M.J., Lilja H., Abrahamsson P.-A., Björk T., Gerdtsson A., Bjartell A. (2012). A Novel automated platform for quantifying the extent of skeletal tumour involvement in prostate cancer patients using the bone scan index. Eur. Urol..

[17] Kolesnikov A., Beyer L., Zhai X., Puigcerver J., Yung J., Gelly S., Houlsby N. (2020). Big transfer (BiT): General visual representation learning. arXiv.

[18] Redmon J., Farhadi A. (2018). YOLO v3: An Incremental Improvement. arXiv.

[19] Bochkovskiy A., Wang C.Y., Liao H.Y.M. (2020). YOLOv4: Optimal Speed and Accuracy of Object Detection. arXiv.

[20] Ren S., He K., Girshick R., Sun J. (2016). Faster R-CNN: Towards Real-Time Object Detection with Region Proposal Networks. arXiv.

[21] He K., Gkioxari G., Dollár P., Girshick R. (2017). Mask R-CNN. arXiv.

[22] Huang Y.J., Chan D.Y., Cheng D.C., Ho Y.J., Tsai P.P., Shen W.C., Chen J.F. (2013). Automated Feature Set Selection and its Application on MCC Identification in Digital Mammograms for Breast Cancer Detection. Sensors.

[23] Cheng D.C., Wu J.F., Kao Y.H., Su C.H., Liu S.H. (2016). Accurate Measurement of Cross-sectional Area of Femoral Artery on MRI Sequences of Transcontinental Ultramarathon Runners Using Optimal Parameters Selection. J. Med. Syst..

[24] https://pjreddie.com/darknet/yolo/.

[25] Zhao Z.Q., Zheng P., Xu S.T., Wu X. (2019). Object detection with deep learning: A review. IEEE Trans. Neural Netw. Learn. Syst..

[26] Hui J. (2018). Object Detection: Speed and Accuracy Comparison (Faster R-CNN, R-FCN, SSD, FPN, RetinaNet and YOLOv3). https://jonathan-hui.medium.com/object-detection-speed-and-accuracy-comparison-faster-r-cnn-r-fcn-ssd-and-yolo-5425656ae359.

[27] Cheng D.C., Liu C.C., Hsieh T.C., Kao C.H. Faster R-CNN in Prostate Cancer Bone Metastasis Identification on Pelvis using Whole Body Bone Scan with Small Database. Proceedings of the 32nd IPPR Conference on Computer Vision, Graphics, and Image Processing.

[28] Shorten C., Khoshgoftaar T.M. (2019). A survey on Image Data Augmentation for Deep Learning. Math. Comput. Simul..

